# Immediate Improvement in Subjective Visual Vertical and Disequilibrium Predicts Resolution of Benign Paroxysmal Positional Vertigo Following Single Canalith Repositioning Maneuver

**DOI:** 10.1097/ONO.0000000000000014

**Published:** 2022-07-18

**Authors:** Christine C. Little, Zachary G. Schwam, Marc Campo, James Gurley, Bryan Hujsak, Maura K. Cosetti, Jennifer Kelly

**Affiliations:** 1 Icahn School of Medicine at Mount Sinai, New York, NY; 2 Department of Otolaryngology, Icahn School of Medicine at Mount Sinai, New York, NY; 3 Physical Therapy Program, Mercy College, Dobbs Ferry, NY; 4 Ear Institute, New York Eye and Ear Infirmary of Mount Sinai, New York, NY.

**Keywords:** Benign paroxysmal positional vertigo, Canalith repositioning maneuver, Modified Clinical Test of Sensory Interaction on Balance, Subjective visual vertical and horizontal, Visual Analog Scale for disequilibrium

## Abstract

**Objective:**

To evaluate whether immediate post-canalith repositioning maneuver (CRM) vestibular changes are predictive of benign paroxysmal positional vertigo (BPPV) resolution.

**Study Design:**

Retrospective cohort study.

**Setting:**

Tertiary referral center.

**Patients:**

Adults (n = 27) with posterior canal BPPV.

**Interventions:**

Single CRM with Frenzel goggles.

**Main Outcome Measures:**

The Visual Analog Scale (VAS) for disequilibrium, the subjective visual vertical (SVV), the subjective visual horizontal (SVH), and the Modified Clinical Test of Sensory Interaction on Balance (mCTSIB) were administered pre- and immediately following single CRM. Dix-Hallpike was performed 1–3 weeks after CRM to assess for BPPV resolution. Pre- and post-treatment vestibular assessments were compared between groups to determine if post-CRM vestibular changes could predict BPPV resolution.

**Results:**

The change in VAS score following CRM treatment was statistically different between patients who responded to CRM treatment (n = 15) and those who did not (n = 12), (–0.07 points versus –2.40 points, respectively; *P* = 0.03). Likewise, a significantly greater improvement in SVV score was observed for CRM responders compared with CRM nonresponders (0.92° versus –0.06°, respectively; *P* = 0.02). Change in SVH and mCTSIB scores did not differ significantly between groups. Additionally, patient age was found to predict outcome of CRM treatment, with older patients more likely to experience persistent BPPV (*P* ≤ 0.01).

**Conclusions:**

Immediate improvement in VAS and SVV scores following CRM may be useful in predicting resolution of BPPV and may assist in directing the timing and need for future interventions. Younger age may have a favorable predictive value for improvement following single CRM.

Benign paroxysmal positional vertigo (BPPV) is a peripheral vestibular disorder characterized by brief attacks of rotary vertigo and nystagmus elicited by changes in head position with respect to gravity (1). BPPV is the most common cause of peripheral vertigo in adults, with a lifetime prevalence of 2.4% and a 1-year incidence rate of 0.6% (2). BPPV is twice as likely to affect women compared with men and predominantly occurs in older adults, with a mean age of onset of 49–57 years across studies (2,3).

The pathophysiology of BPPV is widely accepted to stem from dislodgement of the otoconia from the utricular macula, allowing otoconial debris to enter 1 or more of the semicircular canals. As the head changes position, the gravitational movement of the otoconial debris induces endolymphatic flow and subsequent cupular deviation, resulting in a false sense of rotational movement (4). BPPV can affect any of the 3 semicircular canals, with the posterior canal being the most common site due to its anatomical alignment and gravity-dependent effects on the vestibular labyrinth, followed in incidence by the horizontal canal and, in rare cases, the anterior canal (5).

The majority of BPPV cases are idiopathic in nature (6). Secondary causes of BPPV are those in which a specific causative etiology is identified, such as head trauma, viral illness, or migraine (7). Trauma is estimated to account for 15% of BPPV cases and may occur from a variety of mechanisms including whiplash injuries, direct head trauma, or otologic surgery (6). Diagnosis of posterior canal BPPV is made via torsional, upbeating nystagmus elicited by the Dix-Hallpike maneuver (5). Horizontal canal BPPV is identified by horizontal nystagmus produced by the supine roll test and anterior canal BPPV diagnosed via downbeating positional nystagmus produced by the Dix-Hallpike maneuver (5,8).

Canalith repositioning maneuver (CRM) is the first-line treatment for BPPV and has been shown to be highly effective, with a meta-analysis of 9 controlled clinical trials comprising 505 patients finding an average success rate of 72% after a single session (9). A myriad of CRMs exist and treatment choice depends on the involved semicircular canal (5). The Epley and Semont maneuvers are considered the gold standard treatment for posterior canal BPPV, whereas the Gufoni and Casani maneuvers are recommended for horizontal canal BPPV (5). Although less data exist regarding the use of CRM for anterior canal BPPV, the deep head hanging maneuver and reverse Epley maneuver have been established in the literature as effective treatments (10).

Although CRMs are highly effective in treating BPPV, a significant portion of patients will experience persistent or recurrent BPPV following treatment (5,11). Close follow-up after CRM is therefore recommended for all patients with BPPV, as unresolved BPPV puts patients at increased risk for falls, decreased quality of life, and anxiety (5). However, intensive follow-up care may impose burdensome financial or logistical hurdles and diverts valuable healthcare resources. Developing low-cost, simple outcome measures that can predict the efficacy of CRM treatment can help reduce these costs and promote efficient care (12). The present study aims to evaluate whether immediate changes in vestibular assessments post-CRM treatment can predict whether patients are likely to experience resolution or persistence of their BPPV.

## METHODS

### Study Population

Study participants were recruited from the vestibular rehabilitation department at an academic tertiary referral center. Inclusion criteria for the study included 1) a history consistent with BPPV, as defined by the American Academy of Otolaryngology-Head and Neck Surgery Foundation (AAO-HNSF) as a history of vertigo provoked by changes in head position relative to gravity (5), 2) age >18 years, 3) English speaking, 4) willingness to comply with the protocol and attend all visits, and 5) able to provide written informed consent. Patients were excluded if they had any lower extremity weight-bearing restrictions, were pregnant, or had a history of peripheral neuropathy. Informed consent was obtained by a licensed physical therapist specialized in vestibular rehabilitation if the patient met the above criteria. This study was approved by the New York Eye and Ear Infirmary Institutional Review Board (IRB 15.07).

### Vestibular Function Assessment

Patients underwent comprehensive pre-treatment balance evaluation with 4 different vestibular assessments: the Visual Analog Scale (VAS) for disequilibrium, the subjective visual vertical (SVV) test, the subjective visual horizontal (SVH) test, and the Modified Clinical Test of Sensory Interaction on Balance (mCTSIB).

The VAS is a 0–10 scale used to measure the subjective perception of disequilibrium experienced by each patient. Patients are asked to rate their current level of disequilibrium, where 0 indicates no disequilibrium and 10 indicates disequilibrium to the point of falling. The VAS score for disequilibrium has been validated in the literature, providing consistent results in measuring the effect of therapeutic maneuvers on patients’ perceived vertigo (13).

The SVV and SVH are used to assess otolith function by measuring the capacity to determine true (gravitational) vertical and horizontal, respectively. Normally, what an individual perceives to be a vertical or horizontal line will closely approximate the gravitational line even in the absence of visual landmarks (14). Vestibular dysfunction can result in tilting of the line to one side, thus no longer coinciding with the gravitational vertical or horizontal. The SVV and SVH tests were administered via the bucket method as described in Zwergal et al (15). In the SVV test, patients were asked to sit upright and look into a plastic bucket containing a straight line of glow-in-the-dark tape adhered to the bottom. The outside of the bucket contained a perpendicular line that originated from the center point of a quadrant. The quadrant was divided into degrees, with a zero-line representing true vertical adjusted to the line inside the bucket. The bucket was randomly rotated by the examiner in a clockwise or counterclockwise direction. The bucket was then slowly returned towards the zero-degree position and patients asked to signal when they estimated the line inside the bucket was truly vertical. The degree at which the patient signaled true vertical was recorded by the examiner using the scale on the outside of the bucket. The procedure was administered 5 times each in the clockwise and counterclockwise direction. The deviations were recorded and averaged. The procedure was repeated in an identical manner for the SVH test using a line in the horizontal plane.

The mCTSIB is used to assess sensory interactions required for balance control in patients with vestibular dysfunction. The assessment consists of 4 test conditions: 1) standing on a solid surface with eyes open, 2) standing on a solid surface with eyes closed, 3) standing on a compliant foam surface with eyes open, and 4) standing on a compliant foam surface with eyes closed. The assessment was administered as outlined by Wrisley and Whitney (16). In brief, patients were asked to stand for 30 seconds with their arms crossed over their chest, feet together, and shoes on for each test condition. All participants wore shoes during both pre and post-CRM testing for the mCTSIB, in accordance with prior research demonstrating footwear has no impact on mCTSIB scores (17). The compliant foam surface consisted of an Airex Balance Pad (Airex AG, Inc.), which is widely used in balance testing and has shown reliable, repeatable results in the mCTSIB (18,19). Trials were discontinued if the subjects fell, moved their hands off of their chest, moved their feet, or opened their eyes during a closed-eye trial. Patients who maintained the appropriate test position for the entire 30-second trial received a score of 30. For trials that were discontinued, patients received a score equal to the number of seconds they were able to maintain the test position before cessation of the trial. Patients were allowed 3 attempts for each test condition and the trial scores averaged. Patients who achieved a score of 30 on their first attempt were not required to undergo subsequent trials for that test condition in accordance with standard procedure. Each trial was carried out in succession without rest between conditions.

### Diagnostic Procedure

The diagnosis of posterior canal BPPV was made by a licensed physical therapist using the Dix-Hallpike maneuver, in accordance with AAO-HNSF guidelines (5). A positive test was defined as one that produced subjective vertigo and appropriate nystagmus. Each test was performed on the left and right to determine laterality. A comprehensive history was obtained in order to determine the etiology of BPPV. Patients were classified as having primary (idiopathic) BPPV if no clear etiology could be identified and secondary BPPV if there was a clear history of recent trauma, labyrinthitis, sudden hearing loss, Meniere’s disease, or history of migraines (7).

### Intervention and Evaluation of Treatment Effect

Patients who tested positive for posterior canal BPPV on the Dix-Hallpike maneuver underwent treatment for BPPV with a single session of the Epley maneuver as outlined by the AAO-HNSF guidelines (5). A post-treatment balance assessment was administered within 5 minutes of CRM using identical methods to the pre-treatment balance assessment.

Patients were asked to return to the clinic 1–3 weeks after receiving CRM treatment to assess for the resolution or persistence of BPPV symptoms. Patients were reevaluated using the Dix-Hallpike maneuver by the same physical therapist seen on original diagnostic testing. Participants who tested negative for BPPV as determined by the Dix-Hallpike maneuver were placed into the CRM responder group; participants who tested positive for BPPV were placed into the CRM nonresponder group. Pre- and post-treatment balance assessments were compared between groups to determine if post-CRM balance changes could predict resolution of BPPV.

### Statistical Analysis

Statistical analysis was performed using SAS University Edition (SAS Institute Inc., Cary, NC). Characteristics of patients who responded to CRM treatment were compared with patients who did not respond to treatment using Fisher exact test for categorical variables and the nonparametric Wilcoxon rank-sum test for quantitative variables. A *P* value of ≤ 0.05 was considered statistically significant. Results of the Wilcoxon rank-sum test are presented as mean ± SD.

## RESULTS

### Group Classification and Clinical Characteristics

A total of 27 patients with posterior canal BPPV were included in the study (Table 1). Of these patients, 15 tested negative for BPPV on reevaluation 1–3 weeks post-CRM treatment and were allocated to the CRM responder group. The remaining 12 patients tested positive for BPPV on reevaluation and were allocated to the CRM nonresponder group. Baseline characteristics for each group are presented in Table 1.

**TABLE 1.** Demographic and clinical characteristics of patients who responded to CRM treatment and patients who did not respond to CRM treatment

Characteristic	CRM responders (n = 15)	CRM nonresponders (n = 12)	*P*
Age, y, mean (± SD)	53.1 (± 11.65)	67.6 (± 13.0)	<0.01
Gender, n (%)			0.70
Male	5 (62.5)	3 (37.5)	
Female	10 (52.6)	9 (47.4)	
Side of BPPV, n (%)			0.86
Right	10 (58.8)	7 (41.2)	
Left	2 (40.0)	3 (60.0)	
Bilateral	3 (60.0)	2 (40.0)	
Etiology of BPPV, n (%)			0.09
Idiopathic (primary)	13 (68.4)	6 (31.6)	
Secondary^*a*^	2 (25.0)	6 (75.0)	

^*a*^The secondary causes of BPPV among CRM responders included 1 patient with post-traumatic BPPV and 1 patient with history of migraine. The secondary causes of BPPV among CRM nonresponders included 3 patients with post-traumatic BPPV, 2 patients with history of labyrinthitis, and 1 patient with history of migraine.

BPPV indicates benign paroxysmal positional vertigo; CRM, canalith repositioning maneuver.

A total of 19 patients were diagnosed with idiopathic (primary) BPPV and 8 diagnosed with secondary causes of BPPV including 4 with post-traumatic etiology, 2 with a history of labyrinthitis, and 2 with a history of migraines. A significant difference in the age of BPPV patients was found between CRM responders and CRM nonresponders. Patients who responded to CRM treatment were significantly younger than those who did not respond to treatment (mean age 53.1 versus 67.6, respectively; *P* ≤ 0.01). No statistical difference was found between groups in terms of gender, laterality of BPPV, or etiology of BPPV. Among patients who tested positive for BPPV on reevaluation, none experienced canal conversion and laterality was identical to the initial diagnostic test.

### Response to CRM Therapy by Change in VAS Score

Comparison of pre- and post-treatment VAS scores revealed a statistically significant change in perceived disequilibrium between groups (Table 2). CRM responders experienced a mean change in VAS score of –0.07 points immediately following treatment, compared with patients in the CRM nonresponder group who experienced a mean worsening of perceived disequilibrium by –2.40 points (*P* = 0.03). The absolute pre-treatment and post-treatment VAS scores were not found to differ significantly between groups.

**TABLE 2.** Comparison of VAS, SVV, and SVH score between patients who responded to CRM treatment and patients who did not respond to treatment

Vestibular assessment	CRM responders, mean (± SD)	CRM nonresponders, mean (± SD)	*P*
VAS assessment			
Pre-treatment score	3.80 (± 2.60)	2.50 (± 2.32)	0.20
Post-treatment score	3.87 (± 2.50)	4.90 (± 3.87)	0.48
Change in score	–0.07 (± 1.94)	–2.40 (± 3.17)	0.03
SVV assessment			
Pre-treatment score	2.13 (± 1.13)	1.72 (± 1.16)	0.22
Post-treatment score	1.20 (± 0.96)	1.77 (± 1.0)	0.10
Change in score	0.92 (± 1.24)	–0.06 (± 0.62)	0.02
SVH assessment			
Pre-treatment score	1.39 (± 0.96)	1.26 (± 1.13)	0.46
Post-treatment score	1.22 (± 0.67)	1.45 (± 0.94)	0.61
Change in score	0.17 (± 0.86)	–0.18 (± 1.02)	0.29

CRM indicates canalith repositioning maneuver; SVH, subjective visual horizontal; SVV, subjective visual vertical; VAS, Visual Analog Scale.

### Response to CRM Therapy by Change in SVV and SVH Score

Comparison of pre- and post-treatment SVV scores revealed a statistically significant change in ability to identify true vertical between groups (Table 2). The mean deviation from true vertical improved by 0.92° among patients in the CRM responder group, compared with a mean worsening of –0.06° among patients in the CRM nonresponder group (*P* = 0.02). The change between pre- and post-treatment SVH scores was not statistically significant between CRM responders and CRM nonresponders (Table 2). No significant difference in the absolute pre-treatment or post-treatment SVV or SVH scores was found between groups.

### Response to CRM Therapy by Change in mCTSIB Score

The change between pre- and post-treatment mCTSIB scores, as well as the absolute pre-treatment and post-treatment mCTSIB scores, was not found to differ significantly between CRM responders and CRM nonresponders for any of the 4 conditions assessed by the mCTSIB (Table 3).

**TABLE 3.** Comparison of the 4 conditions of the mCTSIB between patients who responded to CRM treatment and patients who did not respond to treatment

mCTSIB condition	CRM responders, mean (± SD)	CRM nonresponders, mean (± SD)	*P*
Condition 1			
Pre-treatment score	30.00 (± 0)	30.00 (± 0)	1.00
Post-treatment score	30.00 (± 0)	29.75 (± 0.87)	0.30
Change in score	0 (± 0)	0.25 (± 0.87)	0.30
Condition 2			
Pre-treatment score	29.73 (± 1.03)	27.72 (± 7.89)	0.87
Post-treatment score	30.00 (± 0)	24.58 (± 10.32)	0.05
Change in score	–0.27 (± 1.03)	3.14 (± 7.74)	0.08
Condition 3			
Pre-treatment score	30.00 (± 0)	29.36 (± 2.22)	0.30
Post-treatment score	30.00 (± 0)	27.39 (± 8.34)	0.12
Change in score	0 (± 0)	1.97 (± 8.84)	0.53
Condition 4			
Pre-treatment score	22.37 (± 8.94)	20.58 (± 10.40)	0.76
Post-treatment score	23.04 (± 9.19)	18.30 (± 11.98)	0.29
Change in score	–0.67 (± 9.97)	2.28 (± 10.17)	0.38

Condition 1 denotes solid surface with eyes open, condition 2 denotes solid surface with eyes closed, condition 3 denotes foam surface with eyes open, and condition 4 denotes foam surface with eyes closed.

CRM indicates canalith repositioning maneuver; mCTSIB, Modified Clinical Test of Sensory Interaction on Balance.

## DISCUSSION

Although CRMs are highly effective in treating BPPV, an estimated 8%–50% of patients treated with a single session of CRM will experience persistent BPPV (5). BPPV can lead to significant medical costs when managed incorrectly, with >65% of the BPPV population undergoing unnecessary imaging, diagnostic testing, or therapeutic interventions (5). The burdensome financial cost to individuals, difficulty in complying with follow-up care, and impact on quality-of-life warrant better predictive outcomes of BPPV resolution (5,12). Outcome measures that could aid in prognostication on the efficacy of CRM treatment can help reduce these costs, promote efficient care, and improve allocation of healthcare resources.

The present study aimed to evaluate whether immediate changes in 4 different vestibular assessments were predictive of BPPV resolution following single CRM treatment. Results from our study demonstrate that immediate changes in VAS and SVV score following CRM treatment can predict resolution of BPPV. In contrast, the change in SVH and mCTSIB scores provided no prognostic value.

The VAS for disequilibrium is a subjective tool used to measure the current level of perceived vertigo. The VAS is a useful tool for disequilibrium assessment as it is rapid, easy to use, and can overcome cultural or language barriers (20). The VAS for disequilibrium has been shown to have excellent reliability in patients with peripheral vertigo (intraclass correlation coefficient, 0.85–0.96) (21), making it a dependable measure of disequilibrium in patients with BPPV. Previous studies have demonstrated that BPPV patients treated with CRM demonstrate significant improvement in VAS score compared with untreated controls, suggesting that improvements in VAS score may reflect resolution or improvement of BPPV symptoms (22,23).

Adding to this growing body of literature, findings from our study indicate the immediate change between pre- and post-CRM VAS scores can predict which patients are likely to experience resolution or persistence of BPPV. Patients with persistent BPPV experienced a significant worsening of perceived disequilibrium following CRM treatment compared with patients in which BPPV resolved. This finding suggests that patients with a significant worsening of VAS scores following CRM treatment may require closer follow-up for signs of residual BPPV than those with improved or negligible changes in VAS scores.

The SVV test is used to evaluate otolith function by assessing the ability to visually perceive true (gravitational) vertical. BPPV is thought to cause abnormal SVV due to a loss of otoconia, which results in decreased weight on the utricular macula and thus decreased signaling of the utricular receptor (14). Previous studies have demonstrated that patients with BPPV show a small but significant difference in SVV score compared with healthy controls (mean SVV deviation 2.1°–5.75° for BPPV patients versus 0°–1.5° for controls) (24,25). Findings from the present study reveal a significantly greater improvement in SVV score following CRM treatment among patients with resolution of BPPV compared with those with persistent BPPV. This finding indicates that patients with improvement of SVV score may be more likely to experience successful resolution of BPPV and may require less frequent follow-up than those with no improvement or worsening of SVV score. Although the change in SVV score in our study was small (0.92° for CRM responders), it represents a significant change given that the average baseline deviation in SVV for BPPV patients is small.

Our results are in agreement with previous studies that show resolution of BPPV is accompanied by normalization of the SVV (14,26,27), although our study is the first to report the change between pre- and immediate post-CRM SVV scores have a favorable predictive value on resolution of BPPV. Results from Faralli et al (14) found a significant variation between pre- and immediate post-CRM SVV scores in 87% of patients who experienced resolution of BPPV, compared with no significant variation in SVV score for those with persistence of BPPV. Improvement in SVV score following CRM therapy is thought to reflect migration of otolith debris from the semicircular canals into the utricle (14).

Data investigating the effect of CRM treatment on mCTSIB scores among patients with BPPV is inconsistent and studies are limited. In contrast to our study, Vaz et al (28) found that patients with posterior canal BPPV performed significantly better on conditions 2–6 of the CTSIB following Epley maneuver, corresponding to conditions 2, 3, and 4 of the mCTSIB. Furthermore, recent study by Lee et al (29) demonstrated that patients who experienced residual vertigo performed significantly worse on the mCTSIB 2 weeks following CRM treatment compared with those without residual vertigo. However, the study by Lotfi et al (30) found no significant difference in mCTSIB score immediately following CRM treatment among patients with posterior canal BPPV, in accordance with our results.

The mCTSIB is a form of static balance testing that assesses the influence of visual, vestibular, and proprioceptive inputs on postural control (17,31). Importantly, studies have demonstrated that BPPV patients display increased lateral and anterior-posterior sway velocity during static balance testing, even among those with normal balance times (30,32–34). Improvements in sway velocity on static balance testing have been found in BPPV patients following CRM, suggesting sway velocity may be a more sensitive marker of BPPV resolution than balance time (30,33,34). Future studies are needed to determine if alternative methods of static balance testing, such as sway velocity or single leg stance, can serve as predictive markers of BPPV resolution.

In addition to changes in balance, the present study found that age is an important prognostic factor for determining which patients will respond to CRM therapy. Patients who successfully responded to CRM treatment were statistically younger than those who experienced persistent BPPV. This finding is consistent with the literature, with previous studies reporting that older age is a significant risk factor for persistent or recurrent BPPV (35,36). Thus, older patients may require further vestibular rehabilitation even after CRM therapy and may benefit from close follow-up for signs of BPPV recurrence.

Although the present study found no significant difference in BPPV etiology between CRM responders and nonresponders, it is important to note that previous studies have observed CRM treatment to be less effective in patients with secondary causes of BPPV compared with those with idiopathic etiology (5,11). Failure of BPPV etiology to achieve statistical significance in our study may reflect lack of statistical power due to small sample size. Further studies with larger cohorts are needed to definitively determine if BPPV etiology may influence likelihood of CRM efficacy.

The study has several limitations. First, our study population was comprised of patients who underwent single CRM with a trained vestibular rehabilitation physical therapist at an academic tertiary referral center. Further studies are needed to assess whether our results are generalizable to other clinical settings, such as patients treated with multiple CRMs or providers with less experience in vestibular rehabilitation. Second, the long-term effects of CRM treatment on BPPV were not evaluated. Therefore, changes in balance scores can only predict short-term response to CRM therapy and do not predict the long-term effectiveness of treatment or assess the risk of BPPV recurrence. Lastly, studies have shown that the mCTSIB can be sharpened with head movement trials to improve discrimination of vestibular disorders (37,38). The present study did not include sharpening trials out of concern that head movement following CRM treatment could dislodge the repositioned otoconia and negatively impact the efficacy of the CRM. However, inclusion of such trials may have aided in discriminating CRM responders from nonresponders and is an important limitation of our study.

## CONCLUSIONS

Immediate changes in VAS and SVV scores following CRM treatment differ significantly between CRM responders and CRM nonresponders and may be useful in predicting which patients are likely to experience resolution or persistence of BPPV. In contrast, changes in SVH and mCTSIB scores did not differ significantly between groups. Additionally, patient age was found to predict outcome of CRM treatment, with older patients more likely to experience persistent BPPV. Developing outcome measures to predict the efficacy of CRM treatment is critical to minimize the need for follow-up in patients with financial burdens or travel restrictions and promote efficient care. However, further evaluation is needed to confirm whether these prognostic measures reflect long-term resolution of BPPV or are reproducible in other clinical settings.

## FUNDING SOURCES

None declared.

## CONFLICT OF INTEREST

None declared.

## DATA AVAILABILITY STATEMENT

The datasets generated during and/or analyzed during the current study are not publicly available, but are available from the corresponding author on reasonable request.

## References

[1] Imai T Takeda N Ikezono T; Committee for Standards in Diagnosis of Japan Society for Equilibrium Research. Classification, diagnostic criteria and management of benign paroxysmal positional vertigo. Auris Nasus Larynx. 2017;44:1–6. 27174206 10.1016/j.anl.2016.03.013

[2] von Brevern M Radtke A Lezius F. Epidemiology of benign paroxysmal positional vertigo: a population based study. J Neurol Neurosurg Psychiatry. 2007;78:710–715. 17135456 10.1136/jnnp.2006.100420 PMC2117684

[3] Caruso G Nuti D. Epidemiological data from 2270 PPV patients. Audiol Med. 2005;3:7–11.

[4] Nuti D Zee DS Mandalà M. Benign paroxysmal positional vertigo: what we do and do not know. Semin Neurol. 2020;40:49–58. 31935770 10.1055/s-0039-3402733

[5] Bhattacharyya N Gubbels SP Schwartz SR. Clinical practice guideline: benign paroxysmal positional vertigo (update). Otolaryngol Head Neck Surg. 2017;156(3_suppl):S1–S47. 10.1177/0194599816689667 28248609

[6] Nuti D Masini M Mandalà M. Benign paroxysmal positional vertigo and its variants. Handb Clin Neurol. 2016;137:241–256. 27638076 10.1016/B978-0-444-63437-5.00018-2

[7] Riga M Bibas A Xenellis J Korres S. Inner ear disease and benign paroxysmal positional vertigo: a critical review of incidence, clinical characteristics, and management. Int J Otolaryngol. 2011;2011:709469. 21837242 10.1155/2011/709469 PMC3151504

[8] Califano L Salafia F Mazzone S Melillo MG Califano M. Anterior canal BPPV and apogeotropic posterior canal BPPV: two rare forms of vertical canalolithiasis. Acta Otorhinolaryngol Ital. 2014;34:189–197. 24882928 PMC4035840

[9] White J Savvides P Cherian N Oas J. Canalith repositioning for benign paroxysmal positional vertigo. Otol Neurotol. 2005;26:704–710. 16015173 10.1097/01.mao.0000178128.66482.7e

[10] Anagnostou E Kouzi I Spengos K. Diagnosis and treatment of anterior-canal benign paroxysmal positional vertigo: a systematic review. J Clin Neurol. 2015;11:262–267. 26022461 10.3988/jcn.2015.11.3.262 PMC4507381

[11] Kansu L Avci S Yilmaz I Ozluoglu LN. Long-term follow-up of patients with posterior canal benign paroxysmal positional vertigo. Acta Otolaryngol. 2010;130:1009–1012. 20297928 10.3109/00016481003629333

[12] Yu J Meng G Xu S. Association between Dix-Hallpike test parameters and successful repositioning maneuver in posterior semicircular canal benign paroxysmal positional vertigo: a case-control study. Ann Transl Med. 2020;8:286. 32355730 10.21037/atm.2020.03.03 PMC7186607

[13] Toupet M Ferrary E Grayeli AB. Visual analog scale to assess vertigo and dizziness after repositioning maneuvers for benign paroxysmal positional vertigo. J Vestib Res. 2011;21:235–241. 21846956 10.3233/VES-2011-0420

[14] Faralli M Manzari L Panichi R. Subjective visual vertical before and after treatment of a BPPV episode. Auris Nasus Larynx. 2011;38:307–311. 21227610 10.1016/j.anl.2010.10.005

[15] Zwergal A Rettinger N Frenzel C Dieterich M Brandt T Strupp M. A bucket of static vestibular function. Neurology. 2009;72:1689–1692. 19433743 10.1212/WNL.0b013e3181a55ecf

[16] Wrisley DM Whitney SL. The effect of foot position on the modified clinical test of sensory interaction and balance. Arch Phys Med Rehabil. 2004;85:335–338. 14966723 10.1016/j.apmr.2003.03.005

[17] Whitney SL Wrisley DM. The influence of footwear on timed balance scores of the modified clinical test of sensory interaction and balance. Arch Phys Med Rehabil. 2004;85:439–443. 15031830 10.1016/j.apmr.2003.05.005

[18] Lin CC Roche JL Steed DP. Test-retest reliability of postural stability on two different foam pads. J Nat Sci. 2015;1:e43. 25834842 PMC4378587

[19] Boonsinsukh R Khumnonchai B Saengsirisuwan V Chaikeeree N. The effect of the type of foam pad used in the modified Clinical Test of Sensory Interaction and Balance (mCTSIB) on the accuracy in identifying older adults with fall history. Hong Kong Physiother J. 2020;40:133–143. 33005077 10.1142/S1013702520500134 PMC7526061

[20] Giommetti G Lapenna R Panichi R. Residual dizziness after successful repositioning maneuver for idiopathic benign paroxysmal positional vertigo: a review. Audiol Res. 2017;7:178. 28603599 10.4081/audiores.2017.178 PMC5452628

[21] Kammerlind A-S Bergquist Larsson P Ledin T Skargren E. Reliability of clinical balance tests and subjective ratings in dizziness and disequilibrium. Adv Physiother. 2005;7:96–107.

[22] Uz U Uz D Akdal G Çelik O. Efficacy of epley maneuver on quality of life of elderly patients with subjective BPPV. J Int Adv Otol. 2019;15:420–424. 31846923 10.5152/iao.2019.6483 PMC6937179

[23] Ribeiro KM Freitas RV Ferreira LM Deshpande N Guerra RO. Effects of balance vestibular rehabilitation therapy in elderly with benign paroxysmal positional vertigo: a randomized controlled trial. Disabil Rehabil. 2017;39:1198–1206. 27340939 10.1080/09638288.2016.1190870

[24] Cohen HS Sangi-Haghpeykar H. Subjective visual vertical in vestibular disorders measured with the bucket test. Acta Otolaryngol. 2012;132:850–854. 22667824 10.3109/00016489.2012.668710 PMC3547496

[25] Sapountzi Z Vital V Psillas G. Subjective visual vertical in patients with benign positional paroxysmal vertigo. Hippokratia. 2017;21:159. 30479482 PMC6248000

[26] Gall RM Ireland DJ Robertson DD. Subjective visual vertical in patients with benign paroxysmal positional vertigo. J Otolaryngol. 1999;28:162–165. 10410349

[27] Ferreira MM Ganança MM Caovilla HH. Subjective visual vertical after treatment of benign paroxysmal positional vertigo. Braz J Otorhinolaryngol. 2017;83:659–664. 27746123 10.1016/j.bjorl.2016.08.014 PMC9449175

[28] Vaz DP Gazzola JM Lança SM Dorigueto RS Kasse CA. Clinical and functional aspects of body balance in elderly subjects with benign paroxysmal positional vertigo. Braz J Otorhinolaryngol. 2013;79:150–157. 23670318 10.5935/1808-8694.20130027 PMC9444521

[29] Lee J-Y Lee I-B Kim M-B. Correlation between residual dizziness and modified clinical test of sensory integration and balance in patients with benign paroxysmal positional vertigo. Res Vestibular Sci. 2021;20:93–100.

[30] Lotfi Y Javanbakht M Sayaf M Bakhshi E. Modified clinical test of sensory interaction on balance test use for assessing effectiveness of Epley maneuver in benign paroxysmal positional vertigo patients rehabilitation. Auditory Vestibular Research. 2018;27:12–18.

[31] Cohen H Blatchly CA Gombash LL. A study of the clinical test of sensory interaction and balance. Phys Ther. 1993;73:346–351; discussion 351–354. 8497509 10.1093/ptj/73.6.346

[32] Chang WC Hsu LC Yang YR Wang RY. Balance ability in patients with benign paroxysmal positional vertigo. Otolaryngol Head Neck Surg. 2006;135:534–540. 17011413 10.1016/j.otohns.2005.10.001

[33] Celebisoy N Bayam E Güleç F Köse T Akyürekli O. Balance in posterior and horizontal canal type benign paroxysmal positional vertigo before and after canalith repositioning maneuvers. Gait Posture. 2009;29:520–523. 19138524 10.1016/j.gaitpost.2008.12.002

[34] Chang WC Yang YR Hsu LC Chern CM Wang RY. Balance improvement in patients with benign paroxysmal positional vertigo. Clin Rehabil. 2008;22:338–347. 18390977 10.1177/0269215507082741

[35] Vaduva C Estéban-Sánchez J Sanz-Fernández R Martín-Sanz E. Prevalence and management of post-BPPV residual symptoms. Eur Arch Otorhinolaryngol. 2018;275:1429–1437. 29687182 10.1007/s00405-018-4980-x

[36] Picciotti PM Lucidi D De Corso E Meucci D Sergi B Paludetti G. Comorbidities and recurrence of benign paroxysmal positional vertigo: personal experience. Int J Audiol. 2016;55:279–284. 26963274 10.3109/14992027.2016.1143981

[37] Cohen HS Mulavara AP Peters BT Sangi-Haghpeykar H Bloomberg JJ. Standing balance tests for screening people with vestibular impairments. Laryngoscope. 2014;124:545–550. 23877965 10.1002/lary.24314 PMC3841227

[38] Cohen HS. A review on screening tests for vestibular disorders. J Neurophysiol. 2019;122:81–92. 30995137 10.1152/jn.00819.2018 PMC6689777

